# The difference in sleep, sedentary behaviour, and physical activity between older adults with ‘healthy’ and ‘unhealthy’ cardiometabolic profiles: a cross-sectional compositional data analysis approach

**DOI:** 10.1186/s11556-019-0231-4

**Published:** 2019-12-12

**Authors:** Declan John Ryan, Jorgen Antonin Wullems, Georgina Kate Stebbings, Christopher Ian Morse, Claire Elizabeth Stewart, Gladys Leopoldine Onambele-Pearson

**Affiliations:** 1 Musculoskeletal Sciences and Sport Medicine (MSSM) Research Centre, Department of Exercise and Sport Science, Manchester Metropolitan University, Manchester, M15 6BH UK; 2 Science, University of Northampton, Northampton, Northamptonshire NN1 5PH UK; 3 Musculoskeletal Rehabilitation Research Group, Department of Rehabilitation Sciences, KU Leuven, 3000 Leuven, Flanders Belgium; 4 Research Institute for Sport and Exercise Sciences, Liverpool John Moores University, Liverpool, Merseyside L3 3AF UK

**Keywords:** Accelerometer, Physical behaviour, Ageing, Sedentary, Compositional

## Abstract

**Background:**

Studies have seldom used Compositional Data Analysis (CoDA) to map the effects of sleep, sedentary behaviour, and physical activity on older adults’ cardio-metabolic profiles. This study therefore aimed to illustrate how sleep, sedentary behaviour, and physical activity profiles differ between older adult groups (60–89 years), with ‘low’ compared to those with ‘high’ concentrations of endocrine cardio-metabolic disease risk markers, using CoDA.

**Method:**

Ninety-three participants (55% female) wore a thigh-mounted triaxial accelerometer for seven consecutive free-living days. Accelerometer estimates of daily average hours of engagement in sedentary behaviour (SB), standing, light-intensity physical activity (LIPA), sporadic moderate-vigorous physical activity (sMVPA, accumulated with bouts between 1 and 10 min), 10-min moderate-vigorous physical activity (_10_MVPA, accumulated with bouts ≥10 min), in addition to self-reported sleeping hours were reported. Fasted whole blood concentrations of total cholesterol, triglyceride, glucose, and glycated haemoglobin, and serum lipoprotein lipase (LPL), interleukin-6 (IL-6), and procollagen III N-terminal propeptide were determined.

**Results:**

Triglyceride concentration appeared to be highly dependent on _10_MVPA engagement as the ‘low’ and ‘high’ concentration groups engaged in 48% more and 32% less _10_MVPA, respectively, relative to the geometric mean of the entire study sample. Time-use composition of the ‘low’ LPL group’s engagement in _10_MVPA was 26% less, while the ‘high’ LPL group was 7.9% more, than the entire study sample. Time-use composition of the ‘high’ glucose and glycated haemoglobin groups appeared to be similar as both engaged in more Sleep and SB, and less _10_MVPA compared to the study sample. Participants with a ‘low’ IL-6 concentration engaged in 4.8% more Sleep and 2.7% less _10_MVPA than the entire study sample. Time-use composition of the Total Cholesterol groups was mixed with the ‘low’ concentration group engaging in more Standing and _10_MVPA but less Sleep, SB, LIPA, and sMVPA than the entire study sample.

**Conclusion:**

Older adults should aim to increase 10MVPA engagement to improve lipid profile and decrease SB engagement to improve glucose profile.

## Background

Between 2011 and 2014, medication prescription for the prevention and treatment of circulatory diseases within the adult population increased 2.2 fold in England [1], with 62–95% of adults above the age of 55 years taking at least one prescribed drug per week for the treatment or prevention of any condition [2]. These prescriptions included medications such as lipid lowering, anticoagulant and anti-fibrinolytic drugs; critically, many of these drugs target metabolites that may be regulated by physical activity (PA) interventions [3–5]. For example, in older adult cohorts (65–94 years), 3 х 60 min aerobic sessions per week, for 8 months, at 60–80% of heart rate reserve was sufficient to reduce total cholesterol and triglyceride concentration [6]. Additionally, emerging-evidence also suggests that changes in sedentary behaviour (SB) could affect metabolites, through physiological mechanisms that are different from those for PA (specifically of moderate to vigorous intensity [MVPA]) [7]. In rodent modelling of SB, 6 h of hind limb unloading in rats decreased oxidative skeletal muscle lipoprotein-lipase (LPL) activity by 50% relative to ambulatory controls, whereas, treadmill running (56 m∙min^− 1^, 3.5 h·day^− 1^) did not increase oxidative muscle LPL activity above that of ambulatory controls but did in glycolytic muscle [7]. It is suggested that SB targets post-transcriptional modification of LPL, as LPL mRNA expression remained unchanged during hind limb unloading [7]. Even though SB and PA may act through independent mechanisms in the modulation of cardio-metabolic disease, ultimately, it is the end-point of the relevant metabolite that is of most interest to the end-user. Therefore, it is argued that future studies should consider SB and PA together, not in isolation, to determine their cumulative effects on health status to reflect ‘real-world’ lifestyles.

Previous approaches to Sleep, SB, LIPA, and MVPA analysis have treated these behaviours as independent risk factors for health status [8–10]. Such isolation of the behaviours in statistical analysis is inappropriate, due to the co-dependent nature of time-use variables, and can lead to the under/overestimation of the effects of Sleep, SB, LIPA, and MVPA on health status [11]. These issues can be overcome by using Compositional Data Analysis (CoDA) and presenting, for example, the geometric mean engagement in a behaviour (time-use component) as a standardised score, relative to the geometric mean of all engaged time-use components. This allows the standardised score of the engagement in a particular time-use composition by a sub-group to be presented as a log ratio, relative to the standardised score of the engagement in the same time-use composition intensity by the entire study sample (log ratio = ln[centred geometric mean of sub-group ÷ centred geometric mean of study sample]) [12–14]. CoDA is normally used to compare the time-use composition of two or more groups, relative to the entire study sample and although a relatively new form of analysis in sleep, sedentary behaviour, and physical activity research, CoDA has provided interesting descriptive data. CoDA of the participants from the NHANES 2005–06 cycle suggested those with a relatively low high-density lipid (HDL-C) concentration engaged in 9% less MVPA relative to the overall geometric mean composition. Whereas, those with a healthy HDL-C concentration engaged in 4% more MVPA relative to the overall geometric mean composition [12].

The use of CoDA continues to grow within health research however, the majority of CoDA studies to date have focussed on adolescent to middle-age cohorts [12, 13, 15–17]. Studies that have applied CoDA to older adult cohorts have primarily focussed on adiposity, cardiorespiratory fitness, cholesterol and glucose [18, 19]. Therefore, the aim of this study was to illustrate which time-use composition influences cardio-metabolic parameters in older adults (60–89 years) as cardiovascular-metabolic complications are one of the leading causes of death in this age group [1]. The objective of this study was to determine the difference in the time-use composition between older adults with ‘low’ and ‘high’ endocrine cardio-metabolic disease-risk (ECMDR) profiles using CoDA. It was hypothesised that time-use composition in the ‘high’ ECMDR profile sub-groups would illustrate a greater engagement in SB and a lower engagement in PA relative to the entire study sample geometric mean, which has been shown in previous studies [12, 13].

The current study fits within Research Area 3 of the VIRTUE Framework [11] as it addresses the need to *“determine the prevalence of the optimal time-use balance among populations and specific population subgroups [and] identify the most common unhealthy time-use patterns in different populations”* [11]. This has been possible as our research group previously developed and utilised an accelerometer analysis package, which reported variable suitable for time-use analysis [11], thus fitting also within Research Area 1 of the VIRTUE Framework.

## Methods

### First laboratory visit

The details of the first laboratory visit follows our previously published work [20, 21]. Briefly, older adults (60+ years) who did not suffer from an untreated cardiovascular disease, had not sustained a mobility limiting injury within the last 3 months, did not require a walking aid (e.g. Zimmer frame), were non-diabetic, and had/were not suffering from dementia or similar disease were recruited for the cross-sectional epidemiology study, primarily from local community groups in Cheshire, United Kingdom, between January 2015 and June 2016. Study involvement was approved by the written informed consent of the participant and ethical approval was granted by the University Ethics Subcommittee. Participants provided hard copies of their most recent prescriptions to record which medications may be influencing their cardiovascular-metabolic disease risk, either directly or indirectly. These medications were categorised into blood pressure (BP) medication [mg∙day^− 1^], lipid-lowering medication [mg∙day^− 1^], directly targeting cardiovascular disease (CVD) medication [*n*∙day^− 1^], (in)directly targeting CVD medication [*n*∙day^− 1^], and inflammatory + (in)directly targeting CVD medication [mg∙day^− 1^].

Participants were fitted with a commercially available tri-axial GeneActiv Original accelerometer (Activinsights Ltd., Kimbolton, UK) on the thigh of their dominant leg (anterior aspect at 50% of greater trochanter to femoral condyle distance) using two waterproof adhesive patches (Tegaderm Film, 3 M, North Ryde, Australia). The accelerometer was worn for seven consecutive free-living days, during this time; participants self-reported their sleeping hours by recording the time they turned the lights off to go to sleep at night and what time they woke up to start their day within a sleep diary. The sleep diary was used to estimate the daily average sleeping hours of the participant, whilst the accelerometer estimated the daily average engagement hours for the other time-use components (see below for details).

The accelerometer data was recorded at a 60 Hz frequency and smoothed using 10-s epochs. In-house developed software, The Cheshire Algorithm for Sedentarism (CAS), was used to calculate the time spent engaging in each sleep, sedentary behaviour, and physical activity intensity using Residual G (= √([standard deviation *x* axis]^2^ + [standard deviation *y* axis]^2^ + [standard deviation *z* axis]^2^)) cut-off points, which were developed from a sub-group (*n* = 20) of older adults from the current body of work [20–22]. Behaviours were classified as SB or Standing by CAS if the participant was in the seated/reclined position or upright, respectively [23], and produced a Residual G value, for at least 1 min, below the SB-LIPA cut-off point (0.057 Residual G = 1.5 metabolic equivalent tasks). The Cheshire Algorithm for Sedentarism classified a behaviour as LIPA if the participant was upright and produced a Residual G value, for at least 1 min, that was above the SB-LIPA cut-off point but below the LIPA-MVPA cut-off point (0.216 Residual G = 3 metabolic equivalent tasks). Any movements by the participant, whilst in the upright position, that produced a Residual G value that was equal to or above the LIPA-MVPA cut-off point, for at least 1 min, was classified as MVPA by CAS. Moderate-to-vigorous physical activity was then further classified by CAS into sporadic MVPA (sMVPA, MVPA time accumulated using bouts that were between 1 and 10 continuous minutes), and 10-min MVPA (_10_MVPA, MVPA time accumulated using bouts of 10 continuous minutes or more). This splitting of MVPA was done to align with the 10-min criterion in the 2011 UK physical activity guidelines for MVPA [24]. Inclusion in statistical analyses required at least six 24-h days of data from the accelerometer, as a result three participants were removed from statistical analyses owing to insufficient amount of data.

### Second laboratory visit

#### Whole blood cardio-metabolic analysis

Participants arrived to the laboratory in an overnight (> 10 h) fasted, hydrated state. Where appropriate, participants were asked to refrain from taking medication until testing had been completed. All participants refrained from taking medication prior to the completion of the laboratory tests and all provided a 10 mL venous blood sample. Whole blood analyses of fasting plasma glucose, total cholesterol, and triglycerides were performed immediately using an Accturend Plus (Roche Diagnostics Limited, Welwyn Garden City, UK) monitoring device and Accutrend test strips (Roche Diagnostics Limited, Welwyn Garden City, UK) [25]. Whole blood analysis of glycated haemoglobin (HbA1c) was performed on a sub-group of participants (*n* = 33) using boronate fluorescence quenching (HbA1c 501 device and test cartridges, HemoCue, Ängelholm, Sweden). HemoCue 501 has shown good reliability (Coefficient of Variation [CV] < 5.0%) and validity (Bland-Altman: 4.4 [95%CI -7.3, 16.2] mmol∙mol^− 1^) compared to high performance liquid chromatography ion exchange [26].

Remaining blood samples were stored on crushed ice for less than 2 h before centrifugation at 1687 G for 5 min (Z380, Hermle, Gosheim, Germany). Serum was harvested and stored at − 20 °C in 1.00 mL aliquots (Eppendorf Ltd., Hamburg, Germany) until further analyses.

#### Serum cardio-metabolic analyses

Commercially available enzyme-linked immunosorbent assay kits were used to determine the concentration of serum lipoprotein lipase (LPL) (Cell Biolabs Inc., California, USA), procollagen III N-terminal propeptide (PIIINP) (Biomatik, Delaware, USA), and interleukin-6 (IL-6) (high-sensitivity, Bio-Techne, Minnesota, USA) using a two-fold sample dilution. Manufacturer reported LPL intra-assay CV was 4% whereas it reached 13% in house. For PIIINP, manufacturer sample intra-assay CV was < 10%, which coincided with in house data (6.5–9.6%). IL-6 manufacture intra-assay CV was 7.8% whereas in house it ranged from 7.4–9.2%. ELISA data were derived using a 96-well spectrophotometer (EL808, BioTek, Vermont, USA) connected to a computer running Gen5 v 1.11 software (BioTek, Vermont, USA).

### Statistical analyses

#### Demographics

SPSS version 22 (IBM, New York, USA) was used for statistical analysis. 1 × 5 independent analysis of variance (ANOVA) and bonferroni correction (Kruskall-Wallis and Mann-Whitney U for non-parametric data) was used to see whether participant demographics differed between lustrums of age and determine whether CoDA needed to be performed for age sub-groups or pooled study sample. Data are presented as mean (standard deviation [SD]) or median (interquartile range [IR]) if rules of parametricity are violated. Statistical significance was set at *p* < 0.05.

#### Compositional data analysis

Participants were grouped into ‘low’ or ‘high’ concentration groups for each cardio-metabolic parameter based on whether they were less than or equal to, or above the recognised threshold concentration for the respective cardio-metabolic marker (Table 1). Where threshold concentrations from previous research could not be applied, the median (PIIINP) and mean (HbA1c) concentration of the study sample were used as the threshold. The HbA1c threshold of 6.5% [27] could not be applied to the study sample as the participants did not display diabetic symptoms and therefore every participants’ HbA1c percentages fell below 6.5%. Similarly, the 4780 pg·mL^− 1^ threshold [30] could not be applied as every PIIINP concentration within the current study was below this threshold.

**Table 1:** Cardio-metabolic threshold values used to determine participant groupings into ‘low’ and ‘high’ endocrine concentration

Cardio-metabolic Parameter	Threshold
Glucose	6.0 mmol∙l^− 1^ [27]
Total Cholesterol	5.0 mmol∙l^− 1^ [27]
Triglyceride	1.7 mmol∙l^− 1^ [27]
HbA1c	5.29%^a^
LPL	63.5 pg∙mL^− 1^ [28]
IL-6	2.29 pg∙mL^−1^ [29]
PIIINP	229.215 pg∙mL^-1a^

^a^Mean % HbA1c used as the threshold. Median PIIINP concentration used as the threshold

Using Excel 2013 (Microsoft, Washington, USA), the geometric mean (hrs∙day^−1^) was calculated for each time-use composition for the entire study sample. The grand geometric mean was further calculated for the entire time-use composition data (Sleep + SB + Standing + LIPA + sMVPA +_10_MVPA) of the entire study sample. The study sample data were centred (*cen*^*o*^) by dividing the geometric mean for each time-use composition by the grand geometric mean of the study sample and then dividing by the available time in a day (24 h). These steps were then performed on the cardio-metabolic parameter sub-groups (*cen*^*i*^) (‘low’ and ‘high’). The centred data for each time-use composition, for each sub-group, was divided by the centred data for the respective time-use composition of the entire study sample as a log ratio (ln[*cen*^*i*^ ÷ *cen*^*o*^]). The log ratio represents the sub-group’s engagement in a time-use composition relative to the entire study sample’s standardised engagement in the same time-use composition [12, 13].

#### Handling covariates

Analysis of covariance (ANCOVA) was performed using SPSS version 22 (IBM, New York, USA) to determine whether covariates, previously shown to be associated with cardio-metabolic parameters [31–34] (BP medication [mg∙day^− 1^], lipid-lowering medication [mg∙day^− 1^], directly targeting CVD medication [*n*∙day^− 1^], (in)directly targeting CVD medication [*n*∙day^− 1^], and inflammatory + (in)directly targeting CVD medication [mg∙day^− 1^]), influenced the concentration of the cardio-metabolic parameters within the current study (Table 2). LPL was found to be influenced by inflammatory + (in)directly CVD targeting medication (*p* < 0.05). LPL data were adjusted for the aforementioned covariate and the participants were regrouped before CoDA was performed. No other cardio-metabolic parameters were influenced by the aforementioned covariates.

**Table 2:** Influence of medication on cardio-metabolic parameters

Medication	Cardio-metabolic Parameter						
	Glucose	Triglyceride	Total Cholesterol	HbA1c	LPL	IL-6	PIIINP
BP	0.71	0.93	0.57	0.53	0.14	0.95	0.70
Lipid-lowering	0.36	0.47	0.84	0.63	0.46	0.81	0.53
Directly targeting CVD	0.94	0.06	0.69	0.27	0.89	0.24	0.59
(in)directly targeting CVD	0.64	0.32	0.90	0.68	0.31	0.76	0.35
Inflammatory + (in)directly targeting CVD	0.47	0.92	0.09	0.19	0.00*	0.71	0.88

Data presented as p value. * Bold - Covariate has a significant effect on a cardio-metabolic endocrine parameter concentration (*p*<0.05)

#### Handling ‘essential’ Zeros

Within CoDA, there are rounded zeros, which represent data that could not be measured due to the sensitivity of the equipment used, and there are essential zeros, which represent real values for a parameter. In the current study, essential zeros were common in time-use composition data, as many participants did not engage in _10_MVPA. It is not possible to log transform zeros or calculate geometric means, therefore they need to be accounted for so they still carry weight in the analyses. One method is to remove all the participants who have essential zero data, which has been performed in previous studies [12, 13]. However, given the sample size of the current study (*n* = 93), removal of participants would severely reduce the power of the study and thus, is not deemed an appropriate approach. Therefore, 0.1 was added to every data point, then 0.1 was subtracted from the geometric mean calculations [35] so the essential zeros still carried weight in the analysis.

#### Sleep, sedentary behaviour, and physical activity co-dependence

To determine the co-dependence between time-use components, a variation matrix was used. A variation matrix displays the variance in the study samples’ log-ratios for each time-use composition comparison (Additional file 1: Table S1). A variance close to zero would imply the amounts of time spent in the corresponding behaviours are highly proportional and therefore, suggest a change in engagement of one of those time-use compositions would likely result in a change in engagement in the corresponding time-use composition.

## Results

The demographics of the 93 older adults who participated in the study (73.6 [7.17] years, 55% female) are displayed in Table 3. Notably, there was no difference between lustrum age groups for any of the cardio-metabolic, time-use composition, or covariate parameters. This therefore allowed follow up CoDA with pooled data. For results on the variation matrix of time-use components please see Additional file 1: Table S1.

**Table 3:** Participant demographics displayed per lustrum of age. Data presented as Mean(SD), Median(IR), or geometric mean

Variable	Age Group (years)					
	Pooled	60–65	66–71	72–77	78–83	84+
*n*	93	10	19	31	24	9
Female (%)	55	90	47	54	54	33
*Covariates*						
Directly CVD Meds (*n*∙day^− 1^)^a^	1.17 (1.52)	0.30 (0.48)	0.74 (1.37)	1.39 (1.61)	1.39 (1.53)	1.78 (1.92)
(In)directly CVD Meds (*n*∙day^−1^)^b^	1.62 (1.81)	0.70 (0.67)	1.05 (1.51)	1.81 (1.97)	2.04 (1.92)	2.11 (1.96)
Inn+ (In)directly CVD Meds (mg∙day^−1^)^c^	157.86 (486.49)	293.40 (797.89)	54.71 (73.39)	93.19 (293.99)	136.67 (323.71)	494.88 (1085.38)
BP Meds (mg∙day^−1^)	9.81 (50.79)	3.50 (6.73)	5.47 (13.29)	2.35 (4.89)	8.05 (16.71)	53.00 (154.35)
Lipid-Lowering Meds (mg∙day^−1^)	8.12 (16.58)	11.00 (16.63)	9.41 (15.60)	3.57 (10.96)	13.33 (23.09)	4.44 (13.33)
*Cardio-metabolic Parameters*						
Triglyceride (mmol∙l^−1^)	1.77 (0.81)_m_	2.09 (0.86)_m_	1.51 (0.84)_m_	1.73 (0.68)_m_	1.88 (0.56)_m_	1.89 (0.57)_m_
Total Cholesterol (mmol∙l^−1^)	5.44 (1.39)_m_	6.06 (0.95)	5.64 (1.08)	5.45 (1.07)	5.64 (0.89)	5.27 (0.66)
Glucose (mmol∙l^−1^)	5.72 (1.10)_m_	5.81 (0.81)_m_	5.85 (1.35)_m_	5.60 (1.33)_m_	5.65 (1.38)_m_	6.00 (1.11)_m_
HbA1c (%)	5.29 (0.31)	5.18 (0.22)	5.30 (0.22)	5.34 (0.36)	5.24 (0.33)	5.38 (0.32)
LPL (pg∙mL^−1^)	113.02 (147.80)_m_	174.35 (277.28)_m_	119.13 (127.16)_m_	83.41 (123.97)_m_	117.88 (147.07)_m_	247.58 (332.56)_m_
IL-6 (pg∙mL^−1^)	2.72 (2.77)_m_	3.11 (1.85)_m_	2.39 (2.85)_m_	2.55 (3.29)_m_	2.36 (2.98)_m_	4.14 (6.00)_m_
PIIINP (pg∙mL^−1^)	229.21 (247.97)_m_	205.05 (239.21)_m_	229.21 (208.30)_m_	316.92 (228.81)_m_	167.19 (248.76)_m_	256.99 (406.27)_m_
*sleep, sedentary behaviour, and physical activity parameters*						
Sleep (hrs∙day^−1^)	8.43 (0.77) 8.40_g_	7.88 (0.83) 7.84_g_	8.49 (0.53) 8.48_g_	8.41 (0.73) 8.35_g_	8.62 (0.79) 8.58_g_	8.50 (1.04) 8.45_g_
SB (hrs∙day^−1^)	9.65 (1.33) 9.56_g_	9.81 (1.31) 9.73_g_	9.34 (1.38) 9.25_g_	9.61 (1.36) 9.53_g_	9.59 (1.29) 9.51_g_	10.49 (1.15) 10.44_g_
Standing (hrs∙day^−1^)	1.09 (0.41) 1.02_g_	1.19 (0.30) 1.14_g_	1.16 (0.43) 1.08_g_	1.11 (0.39) 1.03_g_	1.08 (0.43) 1.00_g_	0.89 (0.43) 0.81_g_
LIPA (hrs∙day^−1^)	1.97 (0.63) 1.86_g_	2.11 (0.49) 2.05_g_	1.86 (0.59) 1.78_g_	2.06 (0.63) 1.95_g_	1.99 (0.68) 1.88_g_	1.73 (0.77) 1.57_g_
sMVPA (hrs∙day^−1^)	2.57 (0.64) 2.49_g_	2.68 (0.58) 2.62_g_	2.86 (0.56) 2.80_g_	2.55 (0.59) 2.49_g_	2.53 (0.69) 2.42_g_	2.07 (0.67) 1.96_g_
_10_MVPA (hrs∙day^−1^)	0.08 (0.20)_m_ 0.06_g_	0.06 (0.16)_m_ 0.07_g_	0.15 (0.29)_m_ 0.13_g_	0.08 (0.21)_m_ 0.05_g_	0.09 (0.22)_m_ 0.07_g_	0.07 (0.13)_m_ 0.03_g_
_10_MVPA^d^ (hrs∙day^−1^)	0.48 (0.36) 0.40_g_	0.53 (0.30) 0.43_g_	0.47 (0.22) 0.43_g_	0.54 (0.51) 0.43_g_	0.39 (0.17) 0.35_g_	0.28 (0.09) 0.27_g_

^a^ Participants are currently prescribed an amount of medication that reduces the risk or treats CVD (i.e. statins, warfarin). ^b^ Participants are currently prescribed a medication that may affect the cardiovascular system either directly or as a side effect. ^c^ Participants are currently prescribed a medication that may affect the cardiovascular system either directly or as a side effect, including inflammatory medication. ^d^ Excluding 49 participants who accumulated < 10 min of _10_MVPA in a week (variable not used in CoDA). _m_ Median (IR). _g_ Geometric mean

### Whole blood cardio-metabolic parameters

**Fig. 1:**
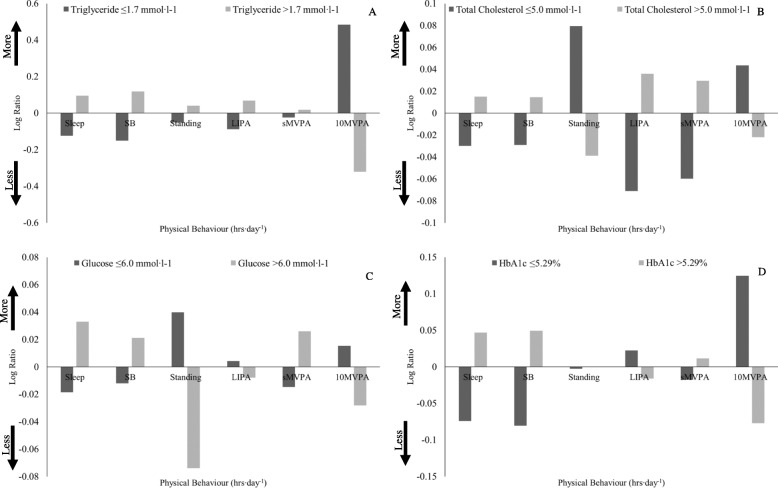
Compositional geometric mean bar plots displaying the difference in time-use composition between ‘low’ and ‘high’ cardio-metabolic parameter sub-groups. **a** triglyceride (n ‘low’: 39, ‘high’: 50) **b** total cholesterol (n ‘low’: 30, ‘high’: 59), **c** glucose (n ‘low’: 57, ‘high’: 32), and **d** HbA1c (n ‘low’: 13, ‘high’: 20). A positive Log Ratio indicates that a group engaged in more of a behaviour, in comparison with the entire study sample. A negative Log Ratio indicates that a group engaged in less of a behaviour, in comparison with the entire study sample

The time-use composition for ‘low’ and ‘high’ whole blood cardio-metabolic parameter sub-groups is displayed in Fig. 1a-d. Triglyceride concentration appears to be highly dependent on _10_MVPA engagement as the ‘low’ triglyceride concentration group engaged in 48% more _10_MVPA relative to the geometric mean of the entire study sample (Fig. 1a). It also suggests that reducing _10_MVPA engagement may result in a change to a ‘high’ triglyceride profile more readily than the opposite. Indeed those with a ‘high’ triglyceride concentration only engaged in 32% less _10_MVPA than the geometric mean of the entire study sample, whereas those with a ‘low’ triglyceride concentration required a 48% greater engagement in _10_MVPA compared to the entire study sample (Fig. 1a). It appears that differences in total cholesterol profile may be influenced by most time-use components as those with a ‘low’ total cholesterol profile engaged in less sleep (3.0%), SB (2.9%), LIPA (7.1%), sMVPA (6.0%), and more standing (7.9%) and _10_MVPA (4.4%) than the entire study sample. The opposite time-use composition was true for the ‘high’ total cholesterol concentration group (Fig. 1b). The composition of the ‘high’ glucose and HbA1c groups appeared to be similar as both engaged in more sleep and SB, and less PA (excluding sMVPA) compared to the entire study sample (Fig. 1c-d).

### Serum cardio-metabolic parameters

**Fig. 2:**
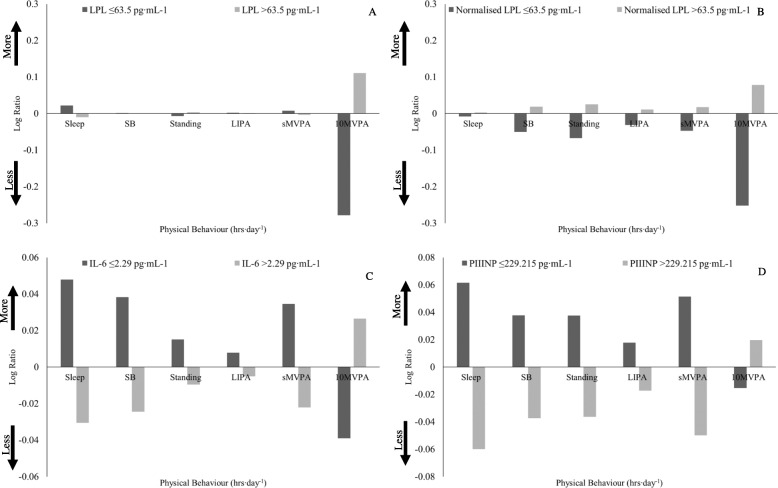
Compositional geometric mean bar plots displaying the difference in time-use composition between ‘low’ and ‘high’ cardio-metabolic parameter sub-groups. **a** LPL (n ‘low’: 26, ‘high’: 57), **b** LPL normalised for inflammatory + CVD (in)directly targeting medication (n ‘low’: 21, ‘high’: 55), **c** IL-6 (n ‘low’: 33, ‘high’: 52), and **d** PIIINP (n ‘low’: 36, ‘high’: 37). A positive Log Ratio indicates that a group engaged in more of a behaviour, in comparison with the entire study sample. A negative Log Ratio indicates that a group engaged in less of a behaviour, in comparison with the entire study sample

Before correcting for covariate effects, LPL was heavily influenced by _10_MVPA, with a large difference in engagement compared to the entire study sample (‘low’ LPL: 27% less _10_MVPA, ‘high’ LPL: 11% more _10_MVPA) (Fig. 2a). Whereas the difference from the entire study sample for the other time-use components did not exceed 2.2% (Fig. 2a). Following normalisation for inflammatory + (in)directly targeting CVD medication, the ‘low’ LPL group’s engagement in _10_MVPA was 26% less while the ‘high’ LPL group was 7.9% more than the entire study sample (Fig. 2b). Sleep appeared to be a main determinant of IL-6 concentration as those in the ‘low’ and ‘high’ IL-6 group engaged in 4.8% more and 3.1% less sleep compared to the entire study sample, respectively. Whereas the other time-use components had a lower difference in engagement compared to the entire study sample (Fig. 2c). The results suggest that bouts of MVPA above 10 mins are sufficient to stimulate an inflammatory response as the ‘high’ IL-6 group engaged in 2.7% more _10_MVPA and 2.2% less sMVPA compared to the entire study sample (Fig. 2c). PIIINP followed a similar composition to IL-6, with sleep displaying the greatest difference from the entire study sample in both ‘low’ (6.2%) and ‘high’ (5.9%) groups compared to the other time-use components.

## Discussion

The findings of this study confirmed our hypothesis as time-use composition in the ‘high’ ECMDR sub-group illustrated a greater engagement in SB and a lower engagement in PA relative to the entire study sample geometric mean. Whereas the ‘low’ ECMDR sub-group engage in more PA and less SB relative to the entire study sample geometric mean which, in agreement with previous studies [12, 13]. However, the hypothesis was not confirmed for IL-6 and PIIINP (when a median grouping threshold was used), as participants within the ‘high’ sub-groups in fact engaged in less SB and more PA.

### Lipoprotein – LPL Axis

Lipoprotein lipase was one of the first identified cardio-metabolic markers to illustrate “independent” effects of SB and MVPA [7]. It was suggested that prolonged SB targets oxidative skeletal muscle LPL activity whereas; MVPA appears to primarily target glycolytic muscle LPL activity [7]. LPL is responsible for the hydrolysis of triglyceride into glycerol and fatty acid. Pre-heparin serum LPL (measured in the current study) primarily represents inactive LPL as a dimer bound to isolated remnant lipoproteins [36], which, in the presence of active LPL, augments triglyceride hydrolysis and the uptake of very low-density lipoproteins and cholesterol esters [37]. Therefore, reduced serum LPL concentration, similar to muscle LPL activity, may lead to increased circulating triglyceride concentration [38]. The comparison of time-use composition between triglyceride and LPL groups supports the LPL-triglyceride-PA complex as those in the ‘high’ LPL concentration and ‘low’ triglyceride concentration groups both displayed a greater engagement in _10_MVPA (7.8 and 48.4%, respectively) compared to the entire study sample. In addition, _10_MVPA had the greatest difference from the entire study sample, compared to the other time-use components, for both LPL and triglyceride, suggesting that these cardio-metabolic markers are influenced more by _10_MVPA rather than any other time-use composition. This finding supports that of a previous SB break study, which found 30 mins of MVPA (which would be classified as _10_MVPA in the current study) maintained plasma triglyceride concentration (relative to baseline) following ingestion of a high fat meal (35%) [39]. In addition, Engeroff, Füzéki [39] reported that short bouts of MVPA (representing sMVPA in the current study) were not sufficient to prevent an increase in triglyceride concentration following meal ingestion. This was also notable in the present study, as engagement in sMVPA in the ‘low’ and ‘high’ triglyceride groups only deviated 2.3 and 1.9% from the entire study sample, respectively, suggesting that sMVPA has little influence on circulating triglyceride levels.

The pattern of time-use composition for total cholesterol is not as clear within the current study. This is likely due to total cholesterol containing lipoproteins that have opposite responses to inactivity. Both triglyceride and low-density lipids (LDL-C) increase in concentration, whereas high-density lipids (HDL-C) decrease in concentration, during detraining [38]. Therefore, it is difficult to ascertain whether/which behaviour(s) are affecting LDL-C and HDL-C profile. Future research, should conduct CoDA with total cholesterol segregated into HDL-C and LDL-C to provide a more precise understand of the effects of sleep, sedentary behaviour, and physical activity.

Overall, our results suggests that engagement in _10_MVPA influences triglyceride concentration by possibly targeting LPL pathways. This finding advocates the need for older adults to be ‘physically active’ in terms of attaining sufficient _10_MVPA, as defined in the 2011 UK government PA guidelines, especially as LPL is already reduced in older adults, compared to young adults [40].

### Glucose metabolism

The prevalence of physical inactivity and SB, even in acute episodes, has a marked influence on insulin insensitivity and subsequently on reduced glucose uptake [41–43], predominantly in skeletal muscle tissue [44]. Our results support this concept as those with a ‘high’ glucose concentration engaged in more sleep and SB, and less PA (excluding sMVPA), compared to the entire study sample. This increased circulating glucose concentration is thought to be due to the reduced translocation of glucose transporter type 4 (GLUT4) to the skeletal muscle cell membrane [45] and reduced expression of carbohydrate metabolism genes during bouts of reduced muscle contractile activity (Sleep and SB) such as, cytoplasmic dynein light chain 1 (*DYNLL1*) [46] which, plays a role in GLUT4 translocation [47]. Our results may also suggest that habitual higher SB and lower PA engagement can have a chronic effect on glucose homeostasis, as participants with a ‘high’ HbA1c percentage also engaged in more sleep and SB, and less _10_MVPA, compared to the entire study sample (with other PA apparently having little effect on HbA1c), when a mean HbA1c percentage grouping threshold was used. HbA1c represents a 1–3 month average of blood glucose concentration [48] and can be used in the diagnosis of diabetes mellitus if blood tests exceed 6.5%. The results of the current study are consistent with previous findings in older English adult populations (≥ 60 years), which found HbA1c percentage increased as objective SB time increased from 8.45–9.52 h∙day^− 1^ to > 9.52 h∙day^− 1^ (5.8 [0.8], 6.0 [0.8]%, *p* = 0.01, respectively) and reduced (0.13% [95%CI -0.24, − 0.03]) per 0.5 h∙day^− 1^ increase in MVPA engagement [49]. Interestingly, in line with our results, Stamatakis, Davis [49] also reported that LIPA was not associated with HbA1c. Our results therefore support that PA has to be of at least moderate intensity in order to maintain increased insulin sensitivity and subsequently glucose uptake, post-exercise [50].

Overall, the current study suggests that reduced SB and increased PA could lead to acutely reduced blood glucose concentration in older adults. However, to maintain a ‘healthier’ chronic glucose homeostasis, PA may have to be of a moderate-vigorous intensity. Therefore, we urge older adults to minimise SB engagement and attain a ‘physically active’ lifestyle to increase the likelihood of a ‘healthy’ glucose profile.

### Inflammation and vascular stiffness

In older adults, IL-6 serum concentration is greater compared to young adults [51] and is associated with an increased risk of CVD [52]. The current study suggested that a greater engagement in sleep and SB could be beneficial towards the reduction in inflammation as those with a ‘low’ IL-6 concentration engaged in 4.8 and 3.8% more sleep and SB, respectively, compared to the entire study sample. This is in agreement with previous older adult findings, which suggested that IL-6 concentration reduced by 2.0 pg∙mL^− 1^ with a > 1.5 h reduction in total awake time [53]. However, it was previously illustrated that an hour increase in SB could increase IL-6 by 0.24 (95%CI 0.13, 0.35) pg∙mL^− 1^ in older adults [54]. The discrepancies between Henson, Yates [54] and the current study may lie in the type of analysis. Henson, Yates [54] used a multiple linear regression model, which does not account for the influence of other behaviour(s) on IL-6 in the same way that CoDA does, and may under-estimate the magnitude and direction of associations, when all other time-use compositions are not accounted for [15]. It is thought that increases in serum IL-6 is a result of increased IL-6 concentration within the muscle, which occurs during repeated muscular contraction [55]. Therefore, it is possible to postulate that an elevated amount of sleep engagement may be necessary for older adults to manage the inflammatory response, as reduced engagement in sleep is associated with an increase in IL-6 concentration and subsequently increased pain ratings within healthy middle-aged adults [56]. This relationship between pain and IL-6 may also explain why the ‘low’ IL-6 group engage in more SB as qualitative evidence stated that older adults’ main determinant for engaging in SB is to reduce sensations of pain [57]. However, given that those within ‘high’ ECMDR groups (total cholesterol, triglyceride, glucose, and HbA1c) appear to engage in more SB, compared to the entire study sample, within the current study; it would be advised that older adults engage in more sleep than SB to improve IL-6 profile.

Increased PIIINP concentration is a marker of vascular stiffness in older adults [30]. Within sleep, sedentary behaviour, and physical activity research, there is an apparent lack of investigations into changes in PIIINP (excluding resistance training). To the author’s knowledge, only one study exists, which suggested 10-weeks of LIPA and MVPA were not sufficient to cause a change in middle-older adults’ (51–71 years) PIIINP concentration [58]. Our results suggest that those with a ‘low’ PIIINP concentration engage in a longer duration of all time-use compositions (excluding _10_MVPA), most noticeably sleep, compared to the study sample and vice versa for the ‘high’ PIIINP group, when a median concentration grouping threshold is applied. The current study, therefore suggests that future research should examine the associations between sleep, sedentary behaviour, and physical activity and PIIINP in older adults to confirm or refute whether sleep, sedentary behaviour, and physical activity interventions can help reduce vascular stiffness through PIIINP pathways.

### Public health implications

The key findings of this study suggest that older adults who have a healthy cholesterol and glucose profile engage in more _10_MVPA and less SB than the entire study sample, respectively. This adds to the evidence base which supports the inclusion of the 10-min threshold within the UK moderate intensity PA guideline. However, given such a low percentage of older adults achieve the moderate intensity PA guidelines (when measured with accelerometery) [59], our secondary finding offers hope that older adults could be able to improve health status by reducing SB, possibly a more palatable option for the population and recommended in the 2019 Chief Medical Officers’ UK Physical Activity Guidelines [60]. Recently, physical activity guidelines for Canada and Australia have moved to considering 24-h time-use composition for children and young people. As CoDA research continues to grow within adult-older adult populations, it appears likely that there will also be a shift to 24-h guideline, which will provide end-users with a variety of options to improve their health-status and thus partially remove the more common one-size fits all approach physical activity guidance.

There appeared to be a pattern across ECMDR variables where a sub-group who engaged in more _10_MVPA also engaged in less sMVPA and vice versa. It seems unlikely that this apparent pattern between sMVPA and _10_MVPA is due to co-dependence as the variation matrix suggested a low co-dependence. Furthermore, without, any direct insight through an experimental manipulation of the two time-use components, it is difficult to put forward a definitive argument to explain why sMVPA and _10_MVPA appear to have opposing relationships with cardiometabolic health markers. We can only speculate that some markers are upregulated by the HIIT (high intensity interval training) type stimulus (sMVPA) and downregulated by the more continuous type of stimulus (_10_MVPA), whilst other markers show the opposite sensitivity. In our data pooling, we categorised activities by threshold so it may be that the absolute amount of energy utilisation may have been higher within the MVPA spectrum in the sMVPA bouts, and lower within this MVPA spectrum in the _10_MVPA bouts. In this event, the fuel utilisation (hence endocrine) profiles of sMVPA and _10_MVPA would differ significantly [61].

### Study Strengths & Limitations

The strengths of the study include: the use of an ‘objective’, posture recognising accelerometer that utilised older adult-relevant sleep, sedentary behaviour, and physical activity intensity cut-off points from SB through to MVPA. In fact, these cut-off points were developed using a sub-sample of the current study’s participants. Another strength is the use of CoDA, which took into account the co-dependence of sleep, sedentary behaviour, and physical activity; and a third strength, the application of time-use composition within older adult populations, which has seldom been carried out.

Nonetheless, the main limiting factor of the current study is the sample size, which constrained the analyses to descriptive group comparisons rather than the more statistically powered compositional regression analysis. Although, the current study provided descriptive comparisons of the time-use composition of ‘healthy’ and ‘unhealthy’ sub-groups, the findings are limited to our sample of participants. Future research is needed with larger sample sizes, statistical difference testing, and longitudinal data from older adults to provide a comprehensive evidence base for generalisation at a population level. Whilst our study sample was too small to do this, and previous studies similar to ours did not attempt this either [12, 13], we would recommend that future studies using larger sample size should statistically determine the significance of differences between sleep, sedentary behaviour, and physical activity of the healthy compared to unhealthy groups. It is possible that the differences look large but are actually not statistically significant. Such an approach would ideally utilise a modification of the bootstrapping method as used in previous work [14].

## Conclusion

CoDA revealed that all time-use components play a role in the maintenance of cardio-metabolic profile. For a ‘healthy’ lipid profile, our results suggested that older adults should attain a ‘physically active’ status, as _10_MVPA engagement was greater in the ‘high’ LPL concentration group and subsequently greater in the ‘low’ triglyceride concentration group. For glucose homeostasis, the current study recommended that older adults should reduce their engagement in SB by engaging in PA. In addition, it was suggested that being ‘physically active’ may contribute to chronic glucose homeostasis, as shown by the HbA1c results. Finally, a 4.8% (approximately 25 mins·day^− 1^, based on the geometric mean of sleep for our study sample) increase in the amount of sleep engagement may be essential for older adults to reduce inflammation, especially in episodes of pain, which has been associated with increasing IL-6 concentration.

Overall, the current study recommends that older adults should aim to be ‘physically active’ by engaging in prolonged bouts of MVPA. However, in the remaining hours of the day, they should aim to reduce SB (in spite of the apparent benefit for IL-6), where possible (to minimise pain), by engaging in low intensity PA (standing and LIPA), as these behaviours are highly co-dependent.

## Supplementary information

**Additional file 1.** Sleep, Sedentary Behaviour, and Physical Activity Co-dependence.

## Data Availability

Upon acceptance of this manuscript, datasets generated and/or analysed during the current study will be available from the Manchester Metropolitan University Repository. Confirmation of web link will be provided at manuscript acceptance.

## Abbreviations

_10_MVPA: 10-min Moderate – Vigorous Physical Activity
CAS: Cheshire Algorithm for Sedentarism
CoDA: Compositional Data Analysis
CVD: Cardiovascular Disease
ECMDR: Endocrine Cardio-Metabolic Disease-Risk
HbA1c: Glycated Haemoglobin
HDL-C: High Density Lipoprotein
IL-6: Interleukin 6
LDL-C: Low Density Lipoprotein
LIPA: Light Intensity Physical Activity
LPL: Lipoprotein Lipase
PA: Physical Activity
PIIINP: Procollagen III N-terminal Propeptide
SB: Sedentary Behaviour
sMVPA: Sporadic Moderate – Vigorous Physical Activity
