# Correction to: The difference in sleep, sedentary behaviour, and physical activity between older adults with ‘healthy’ and ‘unhealthy’ cardiometabolic profiles: a cross-sectional compositional data analysis approach

**DOI:** 10.1186/s11556-020-0236-z

**Published:** 2020-01-30

**Authors:** Declan John Ryan, Jorgen Antonin Wullems, Georgina Kate Stebbings, Christopher Ian Morse, Claire Elizabeth Stewart, Gladys Leopoldine Onambele-Pearson

**Affiliations:** 1 Department of Exercise and Sport Science, Musculoskeletal Sciences and Sport Medicine (MSSM) Research Centre, Manchester Metropolitan University, Manchester, M15 6BH UK; 2 Science, University of Northampton, Northampton, Northamptonshire NN1 5PH UK; 3 Department of Rehabilitation Sciences, Musculoskeletal Rehabilitation Research Group, KU Leuven, 3000 Leuven, Flanders Belgium; 4 Research Institute for Sport and Exercise Sciences, Liverpool John Moores University, Liverpool, Merseyside L3 3AF UK

**Correction to: European Review of Aging and Physical Activity (2019) 16:25**

**https://doi.org/10.1186/s11556-019-0231-4**

Following publication of the original article [1], the authors reported an error on the content of Availability of data and materials section in their paper. It should be corrected from:

“Upon acceptance of this manuscript, datasets generated and/or analysed during the current study will be available from the Manchester Metropolitan University Repository. Confirmation of web link will be provided at manuscript acceptance.”

to

“Data associated with this publication can be accessed via the Manchester Metropolitan University repository using the following link: https://urldefense.proofpoint.com/v2/url?u=http-3A__e-2Dspace.mmu.ac.uk_624523_&d=DwIGaQ&c=vh6FgFnduejNhPPD0fl_yRaSfZy8CWbWnIf4XJhSqx8&r=J-U_lVgGXMYKEbxyTbwI9N9azQNeNZu2KgOlMMyTps4&m=wPuRk8o0Ra0spVIXwSLiGS-X6j3Pd0Hx0zZgX-lhWZU&s=7ZxsFuAjm7ONZGaY430ueWMctP_uWnug1fruBni7l9I&e=”
