# Supplementary material for: The difference in sleep, sedentary behaviour, and physical activity between older adults with ‘healthy’ and ‘unhealthy’ cardiometabolic profiles: a cross-sectional compositional data analysis approach
Source: Eur Rev Aging Phys Act. 2019 Dec 12;16:25. doi: 10.1186/s11556-019-0231-4 (PMC6909533; doi:10.1186/s11556-019-0231-4)
Supplement: Supplementary file 1 — Additional file 1. Sleep, Sedentary Behaviour, and Physical Activity Co-dependence. [file 11556_2019_231_MOESM1_ESM.docx]

*Additional File 1*

*Sleep, Sedentary Behaviour, and Physical Activity Co-dependence*

The greatest co-dependence existed between sleep against SB, standing against LIPA, and LIPA against sMVPA, thus indicating that changes in one of these time-use components will likely result in a change in engagement for the corresponding time-use component. The lowest co-dependence existed between _10_MVPA and all other time-use components (Additional File 1, table 1), indicating that changes in _10_MVPA engagement will likely not result in changes in other time-use components.

*Table 1. Variation matrix between time-use components.*

|  | **Sleep** | **SB** | **Standing** | **LIPA** | **sMVPA** | **_10_MVPA** |
| --- | --- | --- | --- | --- | --- | --- |
| Sleep |  | 0.03 | 0.20 | 0.15 | 0.10 | 1.88 |
| SB |  |  | 0.24 | 0.19 | 0.15 | 1.93 |
| Standing |  |  |  | 0.09 | 0.16 | 1.95 |
| LIPA |  |  |  |  | 0.09 | 1.85 |
| sMVPA |  |  |  |  |  | 1.62 |
